# Ionizing Radiation as a Source of Oxidative Stress—The Protective Role of Melatonin and Vitamin D

**DOI:** 10.3390/ijms21165804

**Published:** 2020-08-13

**Authors:** Jarosław Nuszkiewicz, Alina Woźniak, Karolina Szewczyk-Golec

**Affiliations:** Department of Medical Biology and Biochemistry, Faculty of Medicine, Ludwik Rydygier Collegium Medicum in Bydgoszcz, Nicolaus Copernicus University in Toruń, 24 Karłowicza St, 85-092 Bydgoszcz, Poland; al1103@cm.umk.pl

**Keywords:** ionizing radiation, melatonin, oxidative stress, radioprotection, reactive oxygen species, vitamin D

## Abstract

Ionizing radiation (IR) has found widespread application in modern medicine, including medical imaging and radiotherapy. As a result, both patients and healthcare professionals are exposed to various IR doses. To minimize the negative side effects of radiation associated with oxidative imbalance, antioxidant therapy has been considered. In this review, studies on the effects of melatonin and vitamin D on radiation-induced oxidative stress are discussed. According to the research data, both substances meet the conditions for use as agents that protect humans against IR-induced tissue damage. Numerous studies have confirmed that melatonin, a hydro- and lipophilic hormone with strong antioxidant properties, can potentially be used as a radioprotectant in humans. Less is known about the radioprotective effects of vitamin D, but the results to date have been promising. Deficiencies in melatonin and vitamin D are common in modern societies and may contribute to the severity of adverse side effects of medical IR exposure. Hence, supporting supplementation with both substances seems to be of first importance. Interestingly, both melatonin and vitamin D have been found to selectively radiosensitise cancer cells, which makes them promising adjuvants in radiotherapy. More research is needed in this area, especially in humans.

## 1. Introduction

The field of radiology dates back to 1895 when the German scientist Wilhelm Konrad Roentgen discovered X-rays [1]. Since then, ionizing radiation (IR) has found wide application in medicine, both in diagnostics and in therapies [2,3,4,5]. The use of medical imaging, especially roentgenodiagnostics and computed tomography, and radiotherapy exposes both patients and medical professionals to the harmful side effects of radiation [2,3]. IR occurs naturally in the environment, having accompanied humanity since its dawn. Its sources are natural radioisotopes found in soil and cosmic rays reaching the Earth’s surface [6,7,8]. This radiation is called background radiation and its value changes with natural conditions [7,9]. The highest background radiation dose values of circa 0.26 Gy/year are observed in Ramsar (Iran) [10]. This dose is 10–100 times higher than the average one, but no greater incidence of cancer or other IR-related diseases is observed in this region [10]. This is due to radiation hormesis, which is an evolutionary adaptation to the presence of background radiation and the development of appropriate repair systems [11].

The mechanism of deleterious IR action is strongly associated with increasing oxidative stress in irradiated tissues [12]. IR is capable of penetrating the cells of living organisms, where it induces the ionization of both organic and inorganic compounds [13,14]. Due to the high water content in cells, radiolysis of water molecules by IR is the main process contributing to the increased formation of reactive oxygen species (ROS) [15,16]. ROS rapidly react with macromolecules, including proteins, nucleic acids and lipids, leading to cell dysfunction and apoptotic cell death [12]. As a result of augmented oxidative stress, not only direct negative side effects, but also ROS-related diseases may develop. Therefore, it is especially important to identify effective and safe prophylactic compounds to protect people from IR damage [4]. Undoubtedly, the substances considered in this type of supporting therapy should demonstrate an ability to counteract excessive oxidative stress.

Recently, attention has been paid to radioprotective properties of two hormones, whose synthesis depends on the specific light wavelengths, namely melatonin and vitamin D [17,18]. Both substances are endogenous compounds, but their deficiencies have been widely described in modern societies [19,20]. Melatonin as a strong direct and indirect antioxidant has been considered a radioprotector since the beginning of the 21st century [21,22,23]. However, animal model and in vitro studies have not been translated into human use yet [23]. Vitamin D, originally associated with bone homeostasis, has been found to perform many regulatory functions, affecting, among other things, the oxidative–antioxidant balance of the body [24,25,26]. Thus, its use to prevent irradiation side effects has also been taken under consideration, but data are limited and require more research.

Taking into account the relevance of the problem, the aim of the current review was to provide new scientific data on the protective effects of melatonin and vitamin D against oxidative damage caused by ionizing radiation. The current state of knowledge, including possible molecular mechanisms of action, is discussed. We hope that our review will be an impetus for further research on the use of both hormones in preventing deleterious side effects of ionizing radiation, especially in the field of human studies.

## 2. Ionizing Radiation as a Source of Reactive Oxygen Species

IR is a form of energy transfer that is able to cause ionization of a material medium while interacting with it [7]. This energy can be transferred by means of electromagnetic waves, including X radiation, gamma radiation and a small range of ultraviolet (UV) radiation with short wavelength and high energy, or through alpha and beta particles [27,28]. Each type of radiation differs in its energy, penetration and biological effects of the exposure. Alpha particles, consisting of two protons and two neutrons, have a short range due to their high mass [29]. There are two types of beta radiation. Beta minus radiation consists of electrons, while beta plus radiation consists of positrons, which are the antimatter counterpart of the electron [30]. Both X and gamma rays are characterized by high penetration and a plate made of lead is needed as an effective shield against them [31]. UV radiation capable of causing ionization has a wavelength in the range of 100–280 nm (UVC) and is absorbed by the atmosphere [28,32].

An important parameter used in dosimetry characterizing IR is linear energy transfer (LET), which determines the average amount of energy lost per unit of length transferred by radiation quanta [33]. High LET values are characteristic of alpha particles, neutrons and cosmic rays (heavy ions) [34]. Alpha particles, compared to other types of radiation, are characterized by shallow penetration, so the radiation energy is deposited at a shorter distance [35]. Neutron radiation and heavy ions, characteristic of cosmic radiation, have a greater range and penetrate deeper than alpha particles [34]. Low LET, typical of beta and gamma types of radiation, involves deposition of energy over a longer distance, causing less damage per distance unit [36].

High LET alpha radiation interacts mainly with molecules on the surface of the tissue by destroying its structure [36]. The most common source of alpha radiation in the environment is one of the natural radon isotopes, namely radon-222 [37]. Despite limited tissue penetration, alpha particles have high relative biological effectiveness. They can cause significant damage, especially in tissues sensitive to alpha particles due to their shallowness, such as bronchial epithelium. This makes radon, as an inhaled residential gas, a significant cause of lung cancer [37]. Characterized by higher penetration, low LET radiation is mainly responsible for the generation of ROS by ionization of atoms [35,38]. It should be noticed that most environmental, occupational and medical IR sources expose people to simultaneous action of different types of radiation. The interaction of low and high LET radiation may lead to increased and more complex biological damage [35].

IR, absorbed by tissues and cells, affects their functioning and structure to various extents, depending on the dose and type of radiation [13,14]. In affected cells, ROS are generated mainly through the radiolysis of water molecules (decay by the action of radiation quanta) or the excitation of water molecules and their decay [15,16,39]. IR can also indirectly influence the oxidative–antioxidant homeostasis by damaging different biomolecules [12]. The altered molecules, such as DNA or proteins responsible for stabilizing the DNA structure, become more susceptible to damage caused by ROS [40,41]. In addition, antioxidants or genes encoding for enzymatic antioxidants can be damaged, which directly increases the oxidative stress [40,41]. A meta-analysis carried out by Einor et al. [42], based on 41 studies concerning various biological matrices, proved that IR, even at low doses, generates ROS.

In biological systems, the state in which the amount of ROS and reactive nitrogen species (RNS) exceeds the physiological ability to maintain homeostasis is called oxidative stress [43,44]. ROS, which are products of excitation and one-, two- and three-electron reduction of the oxygen molecule, are characterized by much greater reactivity than the oxygen in the ground state [45,46]. ROS are a broad concept, including ions, atoms, as well as molecules and radicals such as hydrogen peroxide (H_2_O_2_), singlet oxygen (^1^O_2_), superoxide anion radical (O_2_^•−^) and hydroxyl radical (OH^•^) [46,47]. The hydroxyl radical is the most dangerous for tissues due to its high reactivity and the ability to oxidize many cell components, such as lipids, proteins, carbohydrates and deoxyribonucleic acids [48,49]. As a result of lipid peroxidation, reactive lipid derivatives are formed, which are capable of oxidative damage to other biomolecules [50]. Depending on the fatty acid that undergoes oxidation, trans-4-hydroxy-2-nonenal (4-HNE) and/or malondialdehyde (MDA) are formed as one of the end products, used as markers of the lipid peroxidation level [44]. Oxidative modifications of the protein structure have been observed in many pathophysiological conditions, including the ageing process, as well as apoptosis [51,52]. They lead to a loss of spatial conformation and biological properties, impeded degradation and accumulation of modified protein products, such as protein carbonyl derivatives [51,52]. Oxidative stress causes damage to both mitochondrial and nuclear DNA, which may result in mutation and carcinogenesis. The marker of DNA damage is 8-oxoguanine, a chemical derivative of guanine [53,54]. Oxidative stress is associated with many diseases, including epidemiologically significant diseases of affluence such as cancer [55], cardiovascular disease [56], obesity [57], neurodegenerative diseases [58] and allergic diseases [59].

Oxygen metabolism and the prevalence of ROS have forced living organisms to develop appropriate counteraction mechanisms to minimize the negative effects of oxidative stress [60]. The antioxidant defense system consists of endogenous and exogenous elements. Antioxidant enzymes, which include superoxide dismutases (SODs), catalase (CAT), glutathione peroxidases (GPxs) and glutathione reductase (GR), the enzyme necessary for the proper functioning of GPx, are a part of the endogenous primary enzymatic defense [61,62]. In addition to antioxidant enzymes, reduced glutathione (GSH), a cofactor for GPx, proteins (ferritin, transferrin, ceruloplasmin, albumin), uric acid, melatonin and vitamin D take part in the prevention of excessive oxidative stress [17,63,64,65]. Carotenoids, vitamins A, C, and E, selenium, and polyphenols are the main exogenous antioxidants [66,67]. The cooperation of both endogenous and exogenous antioxidants maintains the oxidative and antioxidant balance, preventing the negative effects of oxidative stress but enabling ROS to perform physiologically important functions as mediators of intercellular communication [68].

In numerous studies, the effect of ionizing radiation on the oxidative stress level has been examined [39,69,70,71]. Different radiation qualities and doses have been used in the experiments during recent years [72,73,74,75]. According to Kang et al. [72], a dose of 2 Gy γ-irradiation at a dose rate of 1.1 Gy/min affected ROS generation in murine splenocyte cell culture. The level of oxidative stress was determined by a method using 2′,7′-dichlorofluorescin diacetate (DCFH-DA), which penetrates inside the cells and is hydrolysed by intracellular esterase into 2′,7′-dichlorofluorescin (DCFH). DCFH reacts with ROS and is converted to highly fluorescent 2′,7′-dichlorodihydrofluorescein (DCF). Fluorescence was assessed 24 h after irradiation and a significant increase in ROS levels was observed as a result of radiation. Similar observations were made by Shaban et al. [75] in a study whose purpose was to investigate the effect of gamma radiation at a dose of 2, 4, 6, 8 and 10 Gy (delivered in four fractions at one-day intervals at a dose rate of 0.5 Gy/min) in male Albino Sprague-Dawley rat testis. The authors examined blood samples and histopathologically evaluated the irradiated tissues. After exposure to IR, increases in MDA, nitric oxide and calcium ion levels were observed, while SOD and CAT activities and GSH concentration decreased. Karimi et al. [73] also described the relationship between gamma radiation at a dose of 15 Gy (at a dose rate of 0.985 Gy/min) and oxidative stress after irradiation of rat lenses. Two days after the exposure to IR, the animals were sacrificed and an increase in MDA concentrations and a decrease in GSH levels were detected in the tested lenses. Rezaeyan et al. [74] irradiated the adult male Sprague-Dawley rat chest area. The applied X-ray at a dose of 18 Gy in one fraction increased oxidative stress 24 h after the exposure through increased MDA levels and decreased SOD activities. It can be summarized that exposure to high doses of IR leads to increased ROS production, enhanced lipid peroxidation and reduced enzymatic antioxidant defense in a dose-, dose-rate- and LET-dependent manner, while low doses of low LET radiation may upregulate antioxidant defense, including the stimulation of GSH synthesis [39]. It has been proven that IR affects ROS and RNS cell metabolism, activating different signaling pathways and disrupting the normal redox system [69,71]. These changes lead to the dysregulation of the activities of cyclooxygenases, lipoxygenases, nitric oxide synthases, and nicotinamide adenine dinucleotide phosphate oxidases, accompanied by mitochondrial dysfunction [69,71]. It is also worth noting that the response to IR is tissue-dependent, with acute damage but fast regeneration for tissues with rapid turnover [70].

## 3. Melatonin—A Circadian Rhythm Regulator with Antioxidant Properties

Melatonin (N-acetyl-5-methoxytryptamine) is a hormone synthesized and secreted mainly by the pineal gland present in the brain of vertebrates [76]. Extrapineal sources of melatonin are localised in bone marrow, skin, platelets, lymphocytes, retina, the gastrointestinal tract, and the Harderian gland [77,78]. It was first isolated from the bovine pineal gland by Aaron Lerner in 1958 [79] and since then researchers have explored new aspects of this hormone.

The pineal gland is an unpaired structure localized between thalamic bodies in the quadrigeminal cistern [80]. In an adult human, this small neuroendocrine gland reaches 5–9 mm in length, 1–5 mm in width, and 3–5 mm in thickness and weighs about 100–180 mg [81]. The substrate for melatonin biosynthesis in pinealocytes is the amino acid containing an indole ring, tryptophan [76,80]. With the tryptophan hydroxylase enzyme (TPH), the tryptophan molecule is converted to 5-hydroxytryptophan (oxitriptan). Then aromatic L-amino acid decarboxylase (AAAD), using pyridoxal phosphate (PLP) as a coenzyme, catalyzes the reaction in which serotonin is formed [82]. 5-hydroxytryptamine (serotonin), a neurotransmitter colloquially called the happiness hormone, is an intermediate for aralkylamine *N*-acetyltransferase (AANAT), which in the presence of acetyl coenzyme A (acetyl CoA) leads to the biosynthesis of N-acetylserotonin (normelatonin) [83]. The last stage of melatonin biosynthesis takes place with the participation of the enzyme acetylserotonin O-methyltransferase (ASMT) and S-adenosyl methionine (SAM), a coenzyme in methylation reactions [84].

Biosynthesis and secretion of melatonin by pinealocytes are regulated by the presence of electromagnetic radiation in the visible light range, especially light with a wavelength of 460–480 nm, which is perceived as blue light [85]. The highest secretion of melatonin is observed between 3:00 a.m. and 4:00 a.m. (with normal circadian rhythms) [86]. The plasma melatonin concentrations during these hours range from 18.5 to 180 pg/mL [87]. Night work and the use of computer screens or smartphones at night, typical of the modern society, lead to reduced melatonin synthesis [20]. In humans, the endogenous master clock, which controls many physiological processes and behavior patterns, is located in the hypothalamic suprachiasmatic nucleus (SCN) [88]. Light reaching intrinsically photosensitive retinal ganglion cells is received by a photopigment sensitive to blue light called melanopsin [88,89]. The signal is transmitted via the retinohypothalamic tract to SCN located above the optic chiasm [80,89]. The SCN has direct connections to other hypothalamic nuclei and the pineal gland [90]. In this way, information sent by SCN regulates melatonin synthesis. Melatonin secreted into the circulatory system affects SCN by feedback, and other tissues by regulating their chronobiology [91]. The *Clock*, *Bmal1*, *Cry1-2*, *Per1-2* genes, whose expression is modulated by melatonin, play an important role in regulating SCN [88,92,93]. The *Bmal1* and *Clock* gene transcription products combine together to form heterodimers, which attach to the promoter region of the *Per* and *Cry* genes to initiate their transcription [94]. In the absence of light, greater transcription of the *Bmal1* and *Clock* genes is observed [94]. In the cytoplasm, PER and CRY proteins combine into a heterodimer, which inhibits further transcription of the genes responsible for their synthesis [92]. Additionally, melatonin is known to attenuate *Cry1* gene expression [93]. The combination of molecular clocks based on the promotion and inhibition of specific gene transcription, and regulation based on external stimuli, namely the presence or lack of blue light, allow the circadian rhythms to function properly. Melatonin acts as a regulator and synchronizer of these processes.

Melatonin is an endocrine, paracrine and autocrine hormone, so it has an effect on tissues distant from the synthesis site, on neighbouring cells, and directly on the cells that synthesize it [82,95]. The action of melatonin occurs through membrane G protein-coupled receptors (MT1, MT2, MT3), but also through nuclear receptors (RZR/RORα) and calmodulin [96,97]. The number of tissues in which MT1 and MT2 receptors have been detected demonstrates the broad spectrum of the compound’s activity, including the liver, kidneys, retina, ovaries, testes, mammary glands, gallbladder, immune cells, cardiovascular system, exocrine pancreas, duodenal enterocytes, brain (hypothalamus, SCN, pituitary), blood vessels, gastrointestinal tract, adipocytes, and skin [98,99,100]. MT1 (MTNR1A), consisting of 350 amino acid residues, couples to pertussis toxin-sensitive G_i_ and toxin-insensitive G_q/11_ proteins, inhibits cAMP response element-binding protein (CREB) phosphorylation, forskolin-stimulated cAMP and protein kinase A signaling, and increases potassium conductance through Kir internally rectifying channels [98,99]. MT2 (MTNR1B), consisting of 362 amino acid residues, inhibits cGMP formation and forskolin-stimulated cAMP production, reduces calcium-dependent dopamine release in the retina and activates protein kinase C (PKC) in the SCN [98,101].

The effect of melatonin is not limited to regulating circadian and seasonal rhythms. Melatonin also modulates the functioning of the immune system [102] and has anti-inflammatory properties [100,103,104,105]. Reduced concentration of melatonin is observed in many pathophysiological conditions and its supplementation may affect the course of disorders, such as neurodegenerative diseases, including Alzheimer’s disease [106,107], primary headache disorders [108], obesity [105,109], diabetes mellitus type 2 [110,111] and hypertension [105,112].

**Figure 1:**
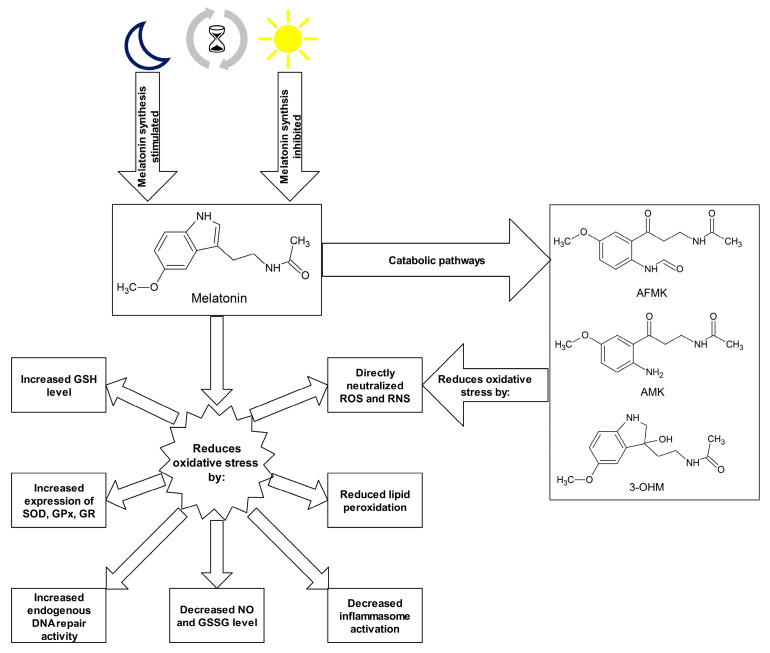
Melatonin and its metabolites as antioxidants. Abbreviations used: 3-OHM—3-hydroxymelatonin, AFMK—N1-acetyl-N2-formyl-5-methoxykynuramine, AMK—N1-acetyl-5-methoxykynuramine, GPx—glutathione peroxidase, GR—glutathione reductase, GSH—glutathione, GSSG—glutathione disulphide, NO—nitric oxide, RNS—reactive nitrogen species, ROS—reactive oxygen species, SOD—superoxide dismutase.

Numerous studies indicate strong antioxidant properties of melatonin [17,77,113,114,115,116]. The molecule can cross the blood–brain barrier and its activity is not limited to the central nervous system (CNS) but it also affects other tissues distant from the site of synthesis [117]. The melatonin is soluble in both water and lipid environments, so it can act as an antioxidant in the aqueous environment inside the cells and in body fluids, as well as in plasma membranes of cells and cell organelles [118]. Research into the antioxidant properties of melatonin has confirmed that this hormone and its metabolites neutralize numerous ROS and RNS molecules, including H_2_O_2_, ^1^O_2_, O^2•−^, peroxynitrite (ONOO-), as well as very reactive OH^•^ [17,119]. Melatonin metabolism products such as N1-acetyl-N2-formyl-5-methoxykynuramine (AFMK), N1-acetyl-5-methoxykynuramine (AMK) and 3-hydroxymelatonin (3-OHM) are also ROS and RNS scavengers [77,120,121]. The antioxidant properties of melatonin are due to its chemical structure, specifically the aromatic indole ring rich in delocalized electrons, a source of electrons in ROS and RNS neutralization reactions [122]. Melatonin may also indirectly affect the oxidative–antioxidant balance, stimulating the expression of genes encoding for some antioxidant enzymes. This effect is observed in the case of SODs, GPxs and GR [123,124]. The effect of melatonin and its chemical derivatives on the oxidoreductive balance is shown in Figure 1.

The study of the effect of melatonin on organisms exposed to IR has been described mainly in animal models [23]. Numerous studies have been conducted on the effect of melatonin pretreatment in the irradiation of specific parts of the body [125,126,127,128,129,130], indicating a reduction in lipid peroxidation, an improvement in enzymatic and non-enzymatic antioxidant defense, stimulation of the DNA damage response, and a reduction in the inflammatory state and histopathological changes. According to Fernandez-Gil et al. [125], melatonin may protect small intestine cells from toxic products formed during radiation therapy used in the oral mucosa. In this experiment, the researchers used adult male Wistar rats, which were given 3% melatonin gel (the total melatonin dose was 45 mg/day for 21 days). The rats were anesthetized prior to irradiation with a dose of 7.5 Gy/day (X-ray) for five consecutive days, where only the oral cavity was irradiated. Melatonin was applied in the oral cavity, starting 48 h before the first irradiation. After the sacrifice of animals, small intestine samples were taken for further analysis. Oral cavity irradiation resulted in small intestinal damage associated with oxidative stress. An increase in lipid peroxidation and nitrite/nitrate was observed, compared to non-irradiated controls. In the melatonin treated group, a significant increase in the activities and protein levels of GPx, GR, SOD2 and a substantial decrease in inflammasome activation in the small intestine were described, compared to the irradiated but non melatonin-treated group. In the Gurses et al. [126] study, Wistar rats were given a 50 mg/kg dose of melatonin (injected intraperitoneally) 15 min prior to irradiation of the anatomical area surrounding the heart. A dose of 18 Gy was used in one fraction. Six months after exposure to radiation, the rats were sacrificed, and histopathological preparations were performed to assess changes in the study and control groups. The use of melatonin prevented the development of vasculitis, and also reduced myocyte necrosis and cardiac fibrosis.

According to the experiments concerning whole body irradiation, melatonin administered both before and after IR exposure increased the survival rate of examined animals, reduced symptoms of acute irradiation disease, decreased histopathological changes, and improved the oxidative–antioxidant balance in the organism [130,131,132,133,134,135,136]. In a mouse model study, Vasin et al. [136] examined the effect of the whole body exposure to IR at a dose of 9.5–10 Gy at a power of 0.077–0.171 Gy/min given in one fraction. Melatonin was dissolved in water at a concentration of 5 mg/L and was administered from 3 to 30 days after the irradiation. The daily dosage of melatonin was changed with the onset of acute radiation sickness to 0.9–1.0 mg/kg and 1.2 mg/kg during recovery. The group of mice treated with melatonin showed less severe symptoms of acute radiation sickness and significantly higher survival was observed within 30 days of irradiation, compared to the control group. At the peak of radiation sickness (12 days after the irradiation), the average number of leukocytes in the group of mice supplemented with melatonin was higher than in the control group by 40%. Similar results were noted in a study conducted by Haddadi et al. [127] on adult male Wistar rats that received melatonin (100 mg/kg b.w.) intraperitoneally 30 min before irradiation and 5 mg/kg per day after irradiation for a maximum of 22 weeks. The animals were anesthetized and sacrificed at 4 and 24 h of irradiation, as well as 1, 3, 8, 16, 20, and 22 weeks after the treatment. The total radiation dose was 22 Gy at a dose rate of 1.8 Gy/min. The authors of the study indicate that the survival of animals from the melatonin-treated group was higher than the control group. In addition, in the melatonin group, lower expression of vascular endothelial growth factor (VEGF) and fewer histopathological changes were shown.

Research data on human studies is very limited. Vijayalaxmi et al. [137] performed an in vitro study on the effect of melatonin on radiated human peripheral blood samples and obtained very promising results. Approximately 15 min before the administration of 300 mg melatonin orally, volunteers gave a blood sample. The next blood collection took place 1 and 2 h after melatonin supplementation. Every blood sample was exposed in vitro to 1 Gy of gamma radiation at a dose rate of 1.087 Gy/min. The lymphocytes were examined to determine the amount of primary DNA damage. A significant increase in melatonin concentration in both serum and leukocytes was observed as early as 1 h after the administration of melatonin. The extent of primary DNA damage was reduced in both blood samples taken 1 and 2 h after melatonin administration, compared to the blood taken before melatonin supplementation. It is worth emphasizing that no negative effects of such a high dose of melatonin (300 mg) were observed. The dose of melatonin given to the subjects in the study is 30 times higher than the safe dose of the substance (10 mg/day) recommended in the treatment of sleep disorders, so further research is crucial to determining the appropriate amount of melatonin needed to protect people from the side effects of irradiation. Table 1 presents the summary of studies on the effects of melatonin on organisms exposed to ionizing radiation.

To complete the picture of the relationship between melatonin and IR, it should be emphasised that melatonin has been found to radiosensitize cancer cells in a selective manner [69,138,139,140]. Melatonin’s ability to sensitize cancer cells to irradiation, along with its radioprotective properties, makes it an ideal adjuvant in radiotherapy [69]. In the case of neck squamous cell carcinoma (HNSCC cell lines), melatonin (0.1, 0.5, 1.0, and 1.5 mM melatonin combined with 8 Gy irradiation) was described to enhance radiation cytotoxicity by stimulating mitochondrial ROS generation, apoptosis and autophagy [138]. It was also observed that melatonin (pretreatment with 1 mmol/L melatonin for 2 h) effectively inhibited cellular proliferation of the human colorectal carcinoma cell line HCT 116, and decreased colony formation rate and cell migration counts following IR exposure (gamma rays, 0–8 Gy) [140]. This effect was associated with activation of the caspase-dependent apoptotic pathway, cell cycle arrest in G2/M, and an impaired DNA double-strand break repair. Moreover, in the study, it was shown that melatonin in combination with IR treatment significantly suppressed tumor cell growth in colorectal tumor xenografts. Analogous results have been confirmed in breast cancer [139]. What remains unestablished is the use of melatonin combined with IR in patients, including the effects of the treatment, the time-lapse between melatonin administration and radiotherapy, as well as the optimal dosage of melatonin in humans exposed to IR. Further patient-based studies, such as pre-clinical and randomized control trials are needed to explain all uncertainties.

## 4. Vitamin D—Function and Antioxidant Effect

Vitamin D is a group of organic chemical compounds belonging to the group secosteroids, among which calcitriol (1,25-dihydroxycholecalciferol) performs the highest biological (hormonal) activity [26,141]. Vitamin D is currently at the center of research interest for many scientists due to its widespread deficiency, reaching about 30–50% on a global scale, especially in older age groups [19,142]. Many scientists have been involved in the research on the discovery and description of vitamin D properties. The largest contribution was made by Sir Edward Mellanby [143], Elmer McCollum [144] and Adolf Windaus [145], who in 1928 received the Nobel Prize for their work on vitamin D [146]. Vitamin D comes from both external sources and from the body’s own synthesis [147]. Two forms of vitamin D are taken with food, namely cholecalciferol (vitamin D_3_) and ergocalciferol (vitamin D_2_) [148]. Fatty fish (such as salmon, mackerel, herring), meat, egg yolks, milk and butter are sources of cholecalciferol, while fungi, yeast and some plants contain ergocalciferol [149,150]. Vitamin D taken from food sources is only a fraction of the daily requirement for this compound [151]. The first stage of calcitriol biosynthesis is the transformation of 7-dehydrocholesterol in the skin under the influence of UV radiation at a wavelength of approximately 290–315 nm (UVB) [152,153]. For that reason, vitamin D is sometimes called the “sunshine vitamin”. Excessive exposure to UV radiation does not cause the formation of toxic amounts of previtamin D because it photoisomerises into two biologically inert products, lumisterol and tachysterol [154]. Previtamin D undergoes spontaneous isomerisation to provitamin D (cholecalciferol) under the influence of body temperature [155]. Then, cholecalciferol both formed in the skin and originating from dietary sources binds to a specific transport protein, vitamin D-binding protein (DBP), and is transported to the liver [156]. Hydroxylation with cytochrome P450 CYP2R1 enzymes occurs in the liver. The product of this reaction, 25-hydroxyvitamin D, binds to DBP and is transported to the kidney for subsequent hydroxylation by the enzyme CYP27B1 [157]. The end product of this pathway is the hormonally active form of vitamin D, calcitriol, which is stored mainly in adipose tissue [158,159]. The vitamin D receptor (VDR) belongs to a subfamily of nuclear receptors that act as transcription factors [160]. VDR is heterodimerized with the retinoid-X receptor (RXR), which causes a change in its spatial conformation. The resulting heterodimer binds to appropriate promoter sites of vitamin D-dependent genes [161]. VDR occurs in almost all cells and tissues, including the skeletal system, cells involved in immune modulation, brain, heart, skin, gonads, prostate, breast and gut [162]. Originally, calcitriol was considered to be associated only with calcium-phosphate metabolism by cooperating with parathyroid hormone and the skeletal system, stimulating the absorption of dietary calcium from the gastrointestinal tract, promoting renal tubular reabsorption of calcium, and inducing the release of calcium from bones [163]. However, the role of vitamin D is known to be much greater and its deficiency is associated not only with diseases of the skeletal system, such as osteomalacia or osteoporosis in adults and rickets in children [164], but also with depression [165], cancer [152], adverse cardiovascular risk profile [166], obesity [24], type 2 diabetes mellitus [25] and autoimmune thyroid disease [167]. The reference vitamin D concentration range is 30–50 ng/mL (75–125 nmol/L) [168,169]. It should be added that this is the level of 25-hydroxyvitamin D, not calcitriol, that is tested because of lower test costs, higher analyte stability and good correlation with the concentration of the hormonally active form in the organism [168,169].

**Figure 2:**
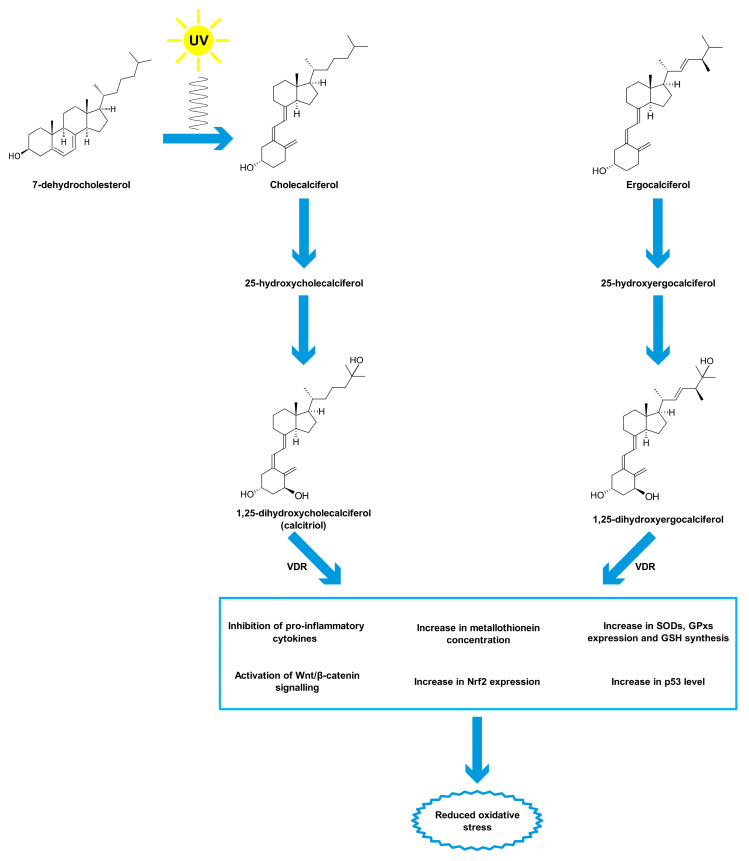
Antioxidant properties of vitamin D. Abbreviations used: GPxs—glutathione peroxidases, GSH—glutathione, Nrf2—nuclear factor-erythroid-2-related factor 2, SODs—superoxide dismutases, VDR—vitamin D receptor.

Vitamin D is thought to have antioxidant properties although involved mechanisms have not been fully described yet and further research is required [18,170]. Vitamin D, acting through its nuclear receptors, can stimulate the expression of genes coding for antioxidant enzymes such as SODs and GPxs [171]. It has also been confirmed that after exposure of the skin to UV radiation, calcitriol and its precursors increase p53 levels, which reduces intracellular ROS [172]. In addition, calcitriol has been shown to induce the synthesis of metallothioneins, which are ROS scavengers [172]. Tang et al. [173] reported that MDA levels negatively correlated with serum vitamin D levels in patients with non-segmental vitiligo. Furthermore, the researchers pointed out that vitamin D protected human melanocytes against ROS by activation of Wnt/β-catenin signaling. In addition, Jain et al. [174] showed a positive link between vitamin D and GSH concentrations, as well as a reduction in the levels of pro-inflammatory cytokines (monocyte chemoattractant protein 1 and interleukin 8), which lead to reduced ROS generation. In this study, U937 monocyte cells were treated with calcitriol at the concentration of 0, 10, and 25 nM for 24 h. Similar results were described by Dzik et al. [175]. In their study, patients, qualified for lumbar spine surgery utilizing static or dynamic implants, were supplemented with 25-hydroxyvitamin D at a daily dose of 3200 IU (equal to 80 µg) for 5 weeks. Vitamin D supplementation to appropriate serum levels reduced oxidative stress in skeletal muscle. The patients with prior vitamin D deficiency showed increases in Cu/ZnSOD and GPx activities in paraspinal muscles after supplementation. Chen et al. [176] tested 10α-hydroxylase knockout mice (1α(OH)ase^-/-^) supplemented with calcitriol at a dose of 1 µg/kg. The authors noted that low calcitriol levels were associated with higher oxidative stress. In addition, calcitriol regulated the expression of nuclear factor-erythroid-2-related factor 2 (Nrf2), which controls antioxidant and detoxification enzymes. In response to reduced ROS levels, SOD2 activity decreased. Sepehrmanesh et al. [177] confirmed that vitamin D supplementation led to a significant increase in GSH concentrations. Patients with major depressive disorder were supplemented with 25-hydroxyvitamin D at a weekly dose of 50,000 IU (equal to 1.25 mg) for 8 weeks. In addition to the increase in the GSH level, there was also a significant increase in total antioxidant capacity (TAC). On the other hand, Barzegari et al. [178] did not observe significant changes in SOD, CAT, and GPx activities, as well as in the MDA and TAC levels, despite a 8-week calcitriol supplementation at 50,000 IU once a week. The study was based on a double-blind, randomized, placebo-controlled clinical trial, involving 50 patients with type 2 diabetes and nephropathy. Undoubtedly, further studies on the antioxidant function of vitamin D are required. The main mechanisms of vitamin D action as an antioxidant are shown in Figure 2.

Considering the broad spectrum of vitamin D action in the organism, it has been identified as a potential protective agent against radiation-induced damage [179]. However, the analysis of the available literature indicates very limited research data on the radioprotective role of vitamin D in the context of IR action. It was observed that the administration of vitamin D_3_ (0.7 μg of vitamin D_3_ or 28 IU/100 g body mass) to chronically irradiated Wistar rats (0.01 Gy per day for 30 days) induced the normalization of carbohydrate metabolism by improving the activities of glycolytic enzymes in erythroid and myeloid bone marrow cells [180]. In the in vitro study of Müller et al. [181], it was found that the cell growth and clonogenic survival of irradiated keratinocytes (cell line HaCaT), pretreated with calcitriol, were significantly increased when compared to the untreated cells after IR exposure. In the experiment, exponentially growing HaCaT cells were irradiated with X-rays (total dose of 0 to 7.5 Gy was delivered with a dose rate of 1 Gy/min). To assess the vitamin D effect, the HaCaT cells were incubated with 10 nmol/L 1α,25(OH)_2_D_3_ for 48 h before irradiation. It was demonstrated that vitamin D improved cell growth and survival, as well as inhibited the radiation-induced up-regulation of adhesion molecule expression on human keratinocytes. These results were confirmed by Langberg et al. [182], who proved that treatment with calcitriol (100 nmol/L 24 h before and for 24–48 h after IR) inhibited caspase-dependent and -independent programmed cell death occurring within 48 h of irradiation, increased the colony formation capacity, attenuated radiation-induced increase in matrix metalloproteinase-9 and mRNA levels in irradiated (4 Gy with a dose rate 2 Gy/min) HaCaT keratinocytes. The same cell line was also the subject of the Trémezaygues et al. [183] experiment. It was observed that the pretreatment of HaCaT-keratinocytes with 1,25(OH)_2_D_3_ (100 nmol/L) over 48 h differentially modulated harmful effects of IR (1–5 Gy) in a dose- and time-dependent manner, indicating a protective effect of vitamin D against relatively low IR (1–2 Gy). A study on the cell culture of human umbilical vein endothelial cells (HUVEC), conducted by Marampon et al. [184], showed the protective effect of vitamin D against damage caused by ROS generated under the influence of IR. Cell cultures were preincubated in a solution with the addition of vitamin D in concentrations of 25, 50, 75 and 100 nmol/L for 24 h, then transferred to the growth medium and irradiated with X-rays (total dose of 0 to 8 Gy was delivered with a dose rate of 1.3 Gy/min). Vitamin D preincubation reduced the amount of ROS by the protection of proliferating and quiescent cells via the regulation of the mitogen-activated protein kinase (MAPK) pathway, prevented apoptosis by activating signal-regulated kinases (ERKs) in proliferating HUVEC, and inhibited p38, associated with ageing in quiescent cells.

As with melatonin, vitamin D and its analogues have been found to selectively radiosensitize cancer cells, including breast and non-small cell lung tumor cells [185,186,187,188,189], which makes it a promising adjuvant in radiotherapy, enhancing the treatment effect and reducing side effects. In irradiated (5 times of 2 Gy administered over a period of 3 days) MCF-7 breast tumor cells, pretreatment with a hormonally active form of vitamin D (100 nmol/L 1,25(OH)_2_D_3_ for 72 h) promoted autophagy, sensitized the cells to IR and suppressed the proliferative recovery occurring after radiation alone [185]. This effect was not observed in the BT474 breast tumor cell line with low-level expression of VDR, suggesting a receptor-mediated action of calcitriol. Moreover, similar responses were not detected in a model of normal human fibroblasts [187]. The promotion of an enhanced response to radiation by 1,25-D_3_ in non-small cell lung cancer cells has been found to be mediated by VDR, tumour protein p53 and AMPK pathways [188]. Normal human bronchial cells and cardiomyocytes were not radiosensitized by vitamin D in this study [188].

Interestingly, it was found that chronic exposure to IR affected the vitamin D_3_ active form level and caused modifications of enzymes involved in vitamin D metabolism [190]. In accordance with this study, Kaminskyi et al. [191] described significantly lower vitamin D concentrations among the populations of radiologically contaminated regions of Chernivtsi oblast due to the Chornobyl catastrophe, compared to those in the uncontaminated Ukrainian cities of Charnivtsi and Vyzhnytsia. Therefore, the deficiency of vitamin D in patients during radiotherapy or in medical professionals chronically exposed to low IR doses should be taken into consideration in further research on the supplementary treatment.

## 5. Conclusions

This review points to the important role of ionizing radiation as an inducer of oxidative stress, which occurs in the pathogenesis and the course of many diseases. ROS are not only generated during medical procedures that require the use of IR but also when the organism is exposed to sunlight and background radiation present in the environment. The endogenous synthesis of two compounds with antioxidant potential described in this paper, namely melatonin and vitamin D, depends on the presence of light (visible or UV). Numerous studies emphasize the role of melatonin as an antioxidant and its protective effects against IR damage. This hormone both directly and indirectly neutralizes ROS. In the case of vitamin D, further experiments are required that could align its antioxidant mechanisms and protection against IR, as previous publications show conflicting findings. As a result of ever-growing use of IR in medicine, more and more people are being exposed to IR at different doses, including several dozen Gy during radiotherapy. Thus, supportive therapies for both patients and medical professionals are of first importance. Synthetic radioprotective compounds have a limited use because they often induce some undesirable side effects, especially at doses required to achieve maximal radioprotection. According to the research data presented in the review, melatonin could be the best candidate for a radioprotectant in people. Less is known about vitamin D. However, the results have been promising so far. The supporting supplementation with both substances seems to be also important in the context of common deficiencies in melatonin and vitamin D in modern societies, which may contribute to the severity of adverse side effects of medical IR exposure. Moreover, both substances have been found to selectively radiosensitize cancer cells, which makes them promising adjuvants for enhancing the anticancer effect of radiotherapy and improving therapeutic outcomes. Thus, in light of existing studies, melatonin and vitamin D are worth considering as agents for protecting professionals exposed to radiation and patients diagnosed or treated with radiation. Nevertheless, more research is needed in this area, especially in humans. Most importantly, appropriate doses of melatonin and vitamin D, effective in protecting against radiation and safe for people, should be established and tested in clinical trials.

## Figures and Tables

**Table 1:** Research on the impact of ionizing radiation on the generation of reactive oxygen species and the radioprotective role of melatonin.

Subjects	Melatonin Dosage (Route of Administration)	Time of Melatonin Administration	Radiation Dosage (Irradiation Area)	Outcomes	Reference
Adult male Sprague-Dawley rats	10 and 20 mg/kg (IP injection)	Immediately before and after irradiation	X-ray radiation of 8 Gy (whole body)	Melatonin reduced the levels of MDA and increased the GSH concentration.	[134]
Adult female Sprague-Dawley rats	30 and 5 mg/kg (IP injection)	30 min prior to irradiation and on the following days of experiment	Gamma radiation of 5 and 8 Gy (total cranial)	Melatonin decreased the formation of late side effects of radiation. Melatonin administration during radiotherapy protected ocular lenses against radiation-induced oxidative injuries.	[129]
Adult male Wistar rats	100 and 5 mg/kg (IP injection)	30 min before irradiation and once a day per after irradiation	Gamma radiation 22 Gy (cervical segment of the spinal cord)	Melatonin increased survival rate and decreased histopathological changes.	[127]
Adult male Wistar rats	50 mg/kg (IP injection)	15 min prior to irradiation	18 Gy (anatomical area of the heart position)	Melatonin prevented the development of vasculitis, reduced myocyte necrosis and cardiac fibrosis.	[126]
Adult male Wistar rats	10, 20, and 10 mg/kg (IP injection)	Before irradiation, just after irradiation and 24h after irradiation	Gamma radiation 8 Gy, twice (whole body and abdominopelvic)	Melatonin administration inhibited primary spermatocyte degeneration.	[130]
Adult male Wistar rats	0.2 mg/day (IP injection)	Once a day for 14 days before irradiation	Gamma radiation 8 Gy (whole body)	Melatonin had a protective effect on suprarenal gland.	[131]
Adult male Wistar rats	5 and 10 mg/kg (IP injection)	30 min before irradiation	Gamma radiation 6 Gy (whole body)	Melatonin decreased hepatic MDA and nitric oxide (NO) levels.	[135]
Adult male Wistar rats	45 mg/day (PO)	Once a day for 21 days before irradiation	X-ray 7.5 Gy/day for five consecutive days (oral cavity)	Melatonin increased the activities and protein levels of GPx, GR, SOD2 and strongly decreased inflammasome activation.	[125]
Adult both sexes Wistar rats	100 mg/kg (IP injection)	For 5 days post radiation	Total dose of 7.2 Gy in two fractions (whole body)	Melatonin reduced MDA level, rates of oedema, necrosis, neuronal degeneration, and vasodilatation.	[133]
Adult male mice	From 0.9–1.0 to 1.2 mg/kg (PO)	From the third day after irradiation	Gamma radiation 9.5–10 Gy (whole body)	Melatonin reduced symptoms of acute radiation sickness, increased survival rate and leukocyte level.	[136]
Adult male Swiss albino mice	0.1 mg/kg/day (PO)	15 consecutive days prior to radiation	Gamma radiation 6, 8 and 10 Gy (whole body)	Melatonin reduced lipid peroxidation, glutathione disulphide (GSSG) level, deficit in the body and organ weight. Melatonin increased GSH level and survival rate.	[132]
Young adult male squirrels	250 mg/kg (SC injection)	Once a day for four weeks before irradiation	X-ray radiation of 2.06 Gy (abdominal, near the splenic region)	Long term melatonin treatment protected the splenocytes and modulated endogenous DNA repair activity.	[128]
In vitro, human blood	300 mg (PO)	1 h before irradiation of blood sample	Gamma radiation 1 Gy (blood sample)	Melatonin reduced primary DNA damage.	[137]

Abbreviations used: IP injection—intraperitoneal injection, PO—oral administration, SC injection—subcutaneous injection.

## Abbreviations

^1^O_2_	singlet oxygen
3-OHM	3-hydroxymelatonin
4-HNE	trans-4-hydroxy-2-nonenal
AAAD	aromatic l-amino acid decarboxylase
AANAT	aralkylamine *N*-acetyltransferase
AFMK	N1-acetyl-N2-formyl-5-methoxykynuramine
AMK	N1-acetyl-5-methoxykynuramine
ASMT	acetylserotonin O-methyltransferase
CAT	catalase
CNS	central nervous system
CREB	cAMP response element-binding protein
DBP	vitamin D-binding protein
DCF	2′,7′-dichlorodihydrofluorescein
DCFH	2′,7′-dichlorofluorescin
DCFH-DA	2′,7′-dichlorofluorescin diacetate
ERKs	signal-regulated kinases
GPx	glutathione peroxidase
GR	glutathione reductase
GSH	glutathione
GSSG	glutathione disulfide
HUVEC	human umbilical vein endothelial cells
IP	intraperitoneal injection
IR	ionizing radiation
LET	linear energy transfer
MAPKs	mitogen-activated protein kinase pathway
MDA	malondialdehyde
Nrf2	nuclear factor-erythroid-2-related factor 2
O_2_^•−^	superoxide anion radical
OH^•^	hydroxyl radical
ONOO-	peroxynitrite
PKC	protein kinase C
PLP	pyridoxal phosphate
PO	oral administration
RNS	reactive nitrogen species
ROS	reactive oxygen species
RXR	retinoid-X receptor
SAM	S-adenosyl methionine
SC	subcutaneous injection
SCN	suprachiasmatic nucleus
SOD	superoxide dismutase
TAC	total antioxidant capacity
TPH	tryptophan hydroxylase
VDR	vitamin D receptor
